# Effects of Selected Pigments on the Properties of Silicone Resin-Based Paints

**DOI:** 10.3390/ma15144961

**Published:** 2022-07-16

**Authors:** Jakub Lisowski, Bolesław Szadkowski, Anna Marzec

**Affiliations:** Institute of Polymer and Dye Technology, Faculty of Chemistry, Lodz University of Technology, Stefanowskiego 16, 90-537 Lodz, Poland; jakub.lisowski@dokt.p.lodz.pl (J.L.); boleslaw.szadkowski@p.lodz.pl (B.S.)

**Keywords:** dry pigments, spinels, acrylic-silicon paints, paint properties

## Abstract

The purpose of this research was to evaluate the impact of selected pigments on the performance of waterborne emulsion paint. Each pigment was incorporated into the paint at 5% *w*/*w*. Density and viscosity measurements as well as the rub-out test were used to test the wet state properties of the colored paint. Wet-scrub, adhesion-to-substrate, water-uptake, vapor-permeability, UV-aging, and other tests were conducted to evaluate effects of the pigments on the dried paint. Bohemian green earth pigment was found to have the most positive effect, as it improved the water resistance of the paint without changing its rheological properties. Therefore, this pigment was selected for further studies, in which the pigment was included as part of the paint formula rather than as a post-additive. The results were satisfactory, confirming the compatibility of the pigment with the formula. However, a slight change in the rheological profile of the paint was observed during tests on a rotational rheometer. This research shows the need for intensive quality control measures while testing alternative formulations, to both enable early detection of negative effects and identify possible improvements.

## 1. Introduction

Waterborne emulsion paints are widely used as exterior coatings in the construction industry. The properties and applications of waterborne emulsion paints have been well documented [1,2,3,4]. They show excellent performance when it comes to durability, water resistance, and flexibility. They are also simple, single-component products that are ready to use. However, the properties of waterborne emulsion paints continue to be improved [5,6,7,8].

Most manufacturers now offer waterborne emulsion paints in a wide range of colors. Colorants are usually added in the form of pigment pastes instead of dry pigments. This method ensures that the pigments have a minimal impact on the coating properties, provided they are not used excessively. However, such interference does not necessarily diminish the properties of the paint. It can maintain or even improve its performance in some areas. There are many different types of colorants, which affect more than just the color of the paint [9,10,11].

Most research focuses only on the properties that are supposed to be changed by the pigment (or other additive). Some studies also consider the ability of cool pigments to reduce surface temperature [12,13], the properties of organic pigments [14,15,16] and spinel pigments [17,18,19], color changes of thermochromic pigments under temperature [20,21,22], or color fading [23]. However, there are few studies testing the impact of pigments on the functional and processing properties of waterborne emulsion paints or coatings [24,25,26]. Mentioned studies provide little research on the overall impact that additives can have on the product.

This work presents the impacts and possible benefits of including dry pigments in coating formulas by testing modified paint for all important application and processing properties. These effects and slight improvements are not usually investigated while testing the properties of new pigments. In the first part of the research, several pigments (green earth, spinel, iron oxide) were added to a premade base of acrylic–silicone paint. Following tests on the liquid paint and hardened coating, one pigment was selected as the most suitable, and a new paint was formulated with the pigment included as a core ingredient. Subsequently, further tests were conducted to determine the properties of the new paint.

Pigments for tests were chosen from different groups, with various reasons to use them in paint. Iron oxide red is a well-known, UV-stable red pigment; green earths are chosen as more economical alternatives to popular cobalt greens; and zinc–iron bronze is a spinel-type pigment (iron and zinc oxide mineral), with potential to have additional positive effects on the product. Green earths have been used as pigments for thousands years [27]. They offer a less expensive alternative to other green pigments. The most popular green pigments—cobalt greens—are among most expensive, reaching prices of €150–200 per kg. Possible alternatives such as Nicosia Green can be purchased for €16 per kg [28]. Spinels have been shown to have beneficial effects in different applications; for example, reducing metallical defects in ceramics [29], or in anti-corrosive, heat-resistant coatings for metal applications [30]. In the present work, it has been shown that Bohemian Green Earth pigment may be successfully used as an effective coloring additive for paint formulation, while it had no major impact on the characteristics of the original paint, including its viscosity, wet-scrub resistance, and rheological properties. This work highlights the importance of extended product testing after any modification, as even minor changes in recipe can lead to differences in product properties.

## 2. Materials and Methods

### 2.1. Raw Materials

A standard waterborne acrylic-silicone facade paint (Atlas Sp. Z o. o., Łódź, Poland) was used as a carrier for the tested pigments. The following pigments were chosen for testing:

- Zinc–iron bronze Fe_2_O_3_ZnO (Kremer Pigmente GmbH & Co., Aischstetten, Germany)—a spinel pigment obtained under high temperature from iron oxides (II) and (III) and zinc oxide, characterized by high heat resistance, high UV resistance, and low absorption of solar radiation through the entire spectrum. It is classified as a “cool” pigment, able to resist surface heating under sunlight.
- Iron red (Kremer Pigmente GmbH & Co., Aischstetten, Germany)—iron oxide α-Fe_2_O_3_, a red pigment, known by the color index PR101.
- Nicosia green (Kremer Pigmente GmbH & Co., Aischstetten, Germany)—a mixture of earth pigments containing compounds of iron, aluminum, magnesium, and potassium, known by the color index PG23.
- Bohemian earth (Kremer Pigmente GmbH & Co., Aischstetten, Germany)—PG23 excavated at a different location, containing additional silicon and calcium compounds (Fe, Al, Si, K, Mg, Ca). The shade and hue of PG23 may vary due to composition differences, depending on the excavation site.

The following commercial pigment pastes were used only as a reference for color aging tests (they were not taken into consideration when testing properties):

- Commercial green GX11—a pigment paste based on cobalt green pigment Co_2_TiO_4_. PG50—a spinel pigment obtained under high temperature from cobalt (II) oxide and titanium (IV) oxide.
- Commercial red RX10—iron red, PR101, in the form of a pigment paste.
- Commercial red RH6—organic red pigment, known by the color index PR254.

The following material was used as reference filler. It was added to the reference sample to maintain equal dry component concentration through the samples:

- P60 (Piotrowice Sp. Z o.o., Piotrowice, Poland)—standard dolomite filler used in the tested paint.

### 2.2. Sample Preparation

Samples were prepared by the addition of 5% w/w of each pigment to the base paint. The pigments were gradually incorporated to the samples using a rotary agitator at a speed of 1000 rpm. Each sample was placed in a sealed container until needed for research. P60 is a neutral paint filler used in the reference sample to equalize the pigment concentration with the other samples. 

### 2.3. Test Methods

The following test methods were applied to determine the effects of each pigment on the material:

- Density measurements were performed using a density cup with a volume of 100.088 cm^3^ to measure the weight of each sample, followed by density calculations according to the formula given in PN-EN ISO 2811-1:2012 [31]:
  $$\rho =\frac{m}{V}$$
  where *m* is sample mass and *V* is volume. Each sample was measured three times. The results were averaged.
- Viscosity measurements were performed according to PN-EN ISO 2555:2011 [32] using a Brookfield (Middleboro, MA, USA) DV-II+ Pro viscometer at 20 rpm with a spindle for highly viscous liquids. Each sample was measured immediately after incorporating the pigment into the paint.
- Gloss measurement was performed using a GLS glossmeter (Gdańsk, Poland) at angles of 60° and 85°, since the base paint is supposed to be matt, according to PN-EN ISO 2813:2014-11 [33].
- The rub-out test was performed by gently rubbing the surface of the applied paint with a fingertip after short time periods (1, 5, and 10 min after application) [34]. The rub-out test was used to analyze the compatibility of the pigment with the material by observing the occurrence of flocculation.
- The wet-scrub resistance test was performed for 200 cycles of scrub on an Elcometer 1720 (Manchester, UK) using a sponge and a 5% solution of sodium dodecylbenzenesulfonate (ABSNa) as a wetting agent. The samples were prepared by the application of a 300 µm thick coating of each paint on a dedicated scrub test substrate. The coatings were conditioned for 28 days at room temperature to ensure complete drying. The dry samples were measured using a thickness meter and placed in the scrub resistance tester. After the test, the samples were rinsed with water and placed in an air dryer at temperature of 50 °C for 7 days. Subsequently, the thicknesses of the coatings was measured again. Scrub resistance class was determined based on loss of coating thickness (PN-EN 13300:2002 standard) [35].
- Adhesion to the substrate was measured using the TQC cross-cut adhesion test (Hadley, UK). Samples were applied to cardboard using a 300-µm applicator and left for 28 days at room temperature to dry out. Next, the surface of each sample was cut vertically and horizontally, with an angle of 90° between cuts. Adhesion to the substrate was determined based on chipping at the incision edges.
- Water absorption tests were conducted according to the PN-C-81521:1976 standard. Two equal layers of paint were applied to a limestone substrate with a 24-h wait between layers. The dimensions of the limestone substrate were 11.5 × 11 × 2.5 cm. The samples were left to dry out for 28 days at room temperature. The edges and sides of the limestone were covered with silicone to seal any areas not covered with paint. Two samples were prepared for each pigment. The samples were conditioned by dipping in water for 72 h, followed by drying in an air dryer in 50 °C for 72 h and resting for 24 h in room temperature. The samples were then weighed and placed in a container filled with water up to half the height of the sides of the samples. The samples were placed on a piece of sponge with the surface covered with paint facing down, to ensure contact with water throughout the test. After 24 h, the samples were weighed again and left to dry at 50 °C for another 24 h. The process was repeated three times. The water uptake factor was calculated according to the formula [36].
  $$W_{24}=\frac{m_{24}-m_{0}}{S\cdot \sqrt{24\;h}}$$
  where *W*_24_ is the water absorbance factor after 24 h, *m*_24_ is the sample mass after 24 h in water, *m*_0_ is the sample mass before dipping in water and *S* is the surface of the sample covered with paint.
- Vapor permeability was measured using the wet-cup method, according to PN-EN ISO 7783:2018-11 [37]. Samples were prepared by applying two equal layers of paint on an oval substrate, with a 24-h wait between layers. The samples were left to dry out for 28 days. Measuring cups were filled with a saturated solution of anhydrous ammonium dihydrogen phosphate, leaving space of about 30 mm between the surface of the solution and the sample. The samples were placed on top of the measuring cups, sealed, and weighed. The edges of each sample were sealed with rubber bands to ensure vapor circulation exclusively through the sample. The cups were placed in a chamber with forced air circulation and weighed every 24 h for 4 days. The linearity of the graph shows that the test has been correctly executed.
- Open time measurements were conducted using a Q-Lab open time tester (Westlake, OH, USA) equipped with a rod for scratching the surface of the sample and a timescale alongside the sample. As the scratching rod moves steadily over the surface, the timescale makes it possible to monitor the time passing. Coatings were applied with a 200-µm applicator on a glass plate. The measurements lasted for 15 min, during which the scratching rod moved through the sample to the point where it no longer damaged the forming film. Each sample was measured separately immediately after application [34].
- Contrast measurements (mating) were conducted using an X-Rite Color i5 (Grand Rapids, MI, USA) spectrophotometer according to PN-EN ISO 2814:2006 [38]. Samples were prepared by applying a 300-µm film of paint on black-white contrast cards. The samples were conditioned for 28 days at room temperature. Mating ability was determined by measuring Lab values over white and black fields and calculating the ΔE values.
- UV color stability was measured using a X-Rite Color i5 (Grand Rapids, MI, USA) spectrophotometer. The lab values were compared with reference samples using ΔE, according to PN-EN ISO 16474-3:2014_02 [39]. The samples were conditioned using a Q-Lab xenotest chamber (Westlake, OH, USA), where they were exposed to xenon arc lamps imitating full spectrum solar irradiance for 28 days at room temperature. Two series of measurements were conducted: ageing for 150 h and aging for 500 h. After that time, the lab values of the samples were measured
- Particle size measurements were conducted using the light scattering method with a Zetasizer device (Malvern, UK). Samples were prepared by mixing 0.1 g of the pigment in 100 g of water. Ultrasound was used to maximize pigment dispersal. The samples were placed in measuring cuvettes and transferred to the Zetasizer device.

## 3. Results and Discussion

### 3.1. Density and Viscosity

**Figure 1:**
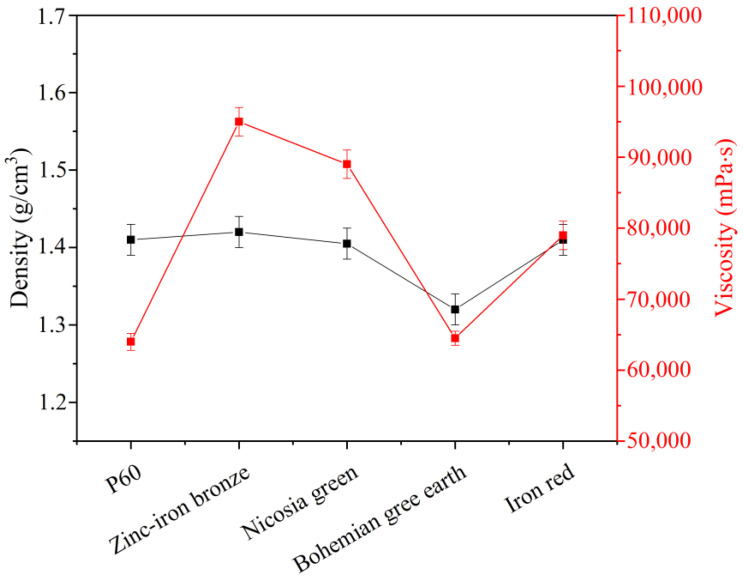
Effects of pigments on paint density and viscosity.

Figure 1 shows the effects of the studied pigments on the density and viscosity of the final products. Viscosity is a main factor in determining the application properties of paints. Waterborne paints are designed to have specific rheological properties, such as sagging resistance or good leveling on walls [40]. It is important to maintain viscosity while changing ingredients. As can be seen in Figure 1, all the pigments showed a significant increase in viscosity (except Bohemian green earth). The increases in viscosity ranged from 20% to 40%, compared to the standard dolomite filler P60. Only Bohemian earth pigment had the same effect on viscosity as the standard filler. Increased viscosity is undesirable for packaging, application, sagging, and leveling. Reduced viscosity is equally undesirable, so modification with Bohemian earth provided the only acceptable outcome. On the other hand, the density of the paint was not significantly increased by the pigments (in the case of zinc–iron bronze and iron red), or as even slightly reduced (in the cases of Nicosia green and Bohemian green earth). This is a positive result, as it could diminish production costs—paints are sold by volumetric measure, whereas raw materials are sold by weigh measure.

### 3.2. Gloss, Rub-Out Test and Substrate Adhesion

None of the chosen pigments affected gloss enough to change the character of the paint (Table 1). As paint is formulated to match a specific type of gloss, new additives should not change the gloss of the product. The second part of table presents the adhesion class and percentage of the exfoliation of the studied pigments. It is important that pigments do not reduce adhesion to the substrate, as this can lead to peeling and the paint falling off. All of the studied pigments were placed in either the first- or second-best categories for substrate adhesion (exfoliation equal 0 means best, class 0, and exfoliation lesser than 5% means second-best class 1), meaning they did not affect the initial properties of the paint. As mentioned in the Methods section, the paint was only applied on cardboard. Although different substrates are known to yield different adhesion results [41], it was only meant to compare the same paint with no difference in binder, so use of one substrate was deemed to be sufficient.

No effect on the compatibility of the pigments was observed in the rub-out test. Each pigment was well incorporated in the paint, and no color differences were observed during application.

### 3.3. Scrub Test

**Figure 2:**
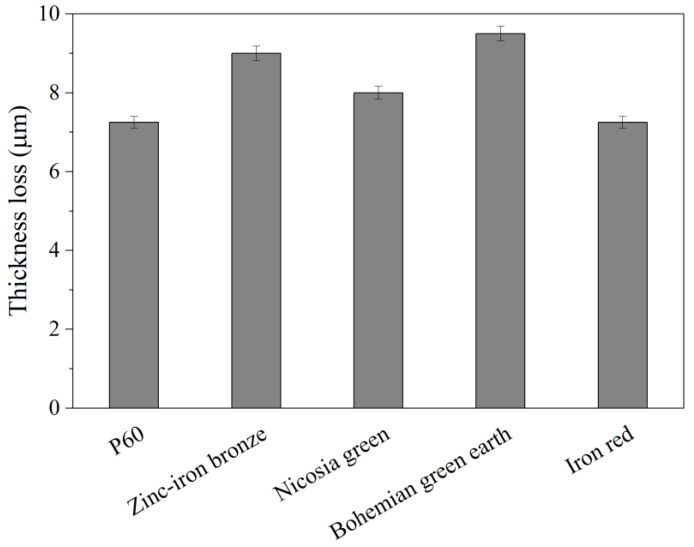
Thickness loss after wet scrub.

As shown in Figure 2, the addition of the studied pigments did not have a major impact on the wet-scrub resistance of the paint. This means that pigment particles were well coated with the polymer and the surface of the coating remained unchanged relative to the standard formulation. It is not uncommon for slight changes in the paint formulation to affect wet-scrub resistance [42]. Therefore, this test should be conducted each time even minor modifications are made. If any of the tested pigments disturb the process of film formation, either chemically or mechanically, this will be observed as a decrease in wet-scrub resistance. All of the tested samples showed reduced thicknesses of between 7 and 10 µm, which is classified as class II scrub resistance following the PN-EN 13300 standard (for thickness loss not greater than 20 µm and not less than 5 µm). Therefore, the tested pigments did not interfere with the process of film formation, compared with the standard formulation. The obtained results of the scrub test seem be in agreement with the data obtained from Zetasizer apparatus (Appendix A Table A1). The size of the pigment particles was close to the size of the standard dolomite filler tested in this study. Particles bigger than standard filler could “stand out” and be removed more easily, causing bigger thickness loss, whereas particles sizes close to those of paint’s fillers did not affect it significantly.

### 3.4. Water Absorption

**Figure 3:**
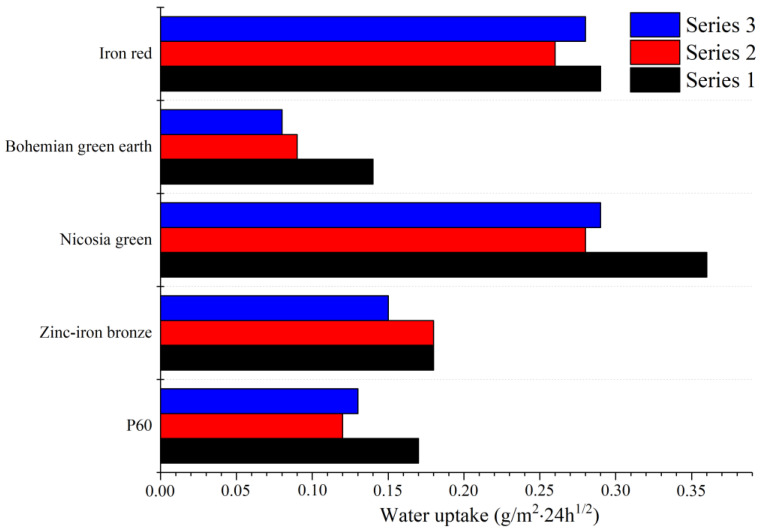
Water uptake after 24 h.

Water absorption was measured three times due to possibility of the emulsifier being rinsed out of sample, which can affect the test outcome. This explains why the second and third series differ quite significantly from first series. The results presented in Figure 3 show that most of the samples can be assigned to class II for water absorption. Bohemian green earth pigment improved the performance of the paint by reducing water uptake to the point of reaching class III water absorption. This improvement may be explained by small-sized pigment particles sealing some of the pores, as well as the presence of the silicon in the structure of the Bohemian green earth pigment. For outdoor applications, reduced water absorption is an important factor, as it restricts moisture transport to the inside of the wall, making it more durable and less vulnerable to cracks and deformations. Bohemian green earth reduced water absorption the most, placing it in highest possible class III according to the PN 76/C-81521 standard. This could have a positive effect on production costs by reducing the use of hydrophobic agents, which are usually expensive.

### 3.5. Vapor Permeability

**Figure 4:**
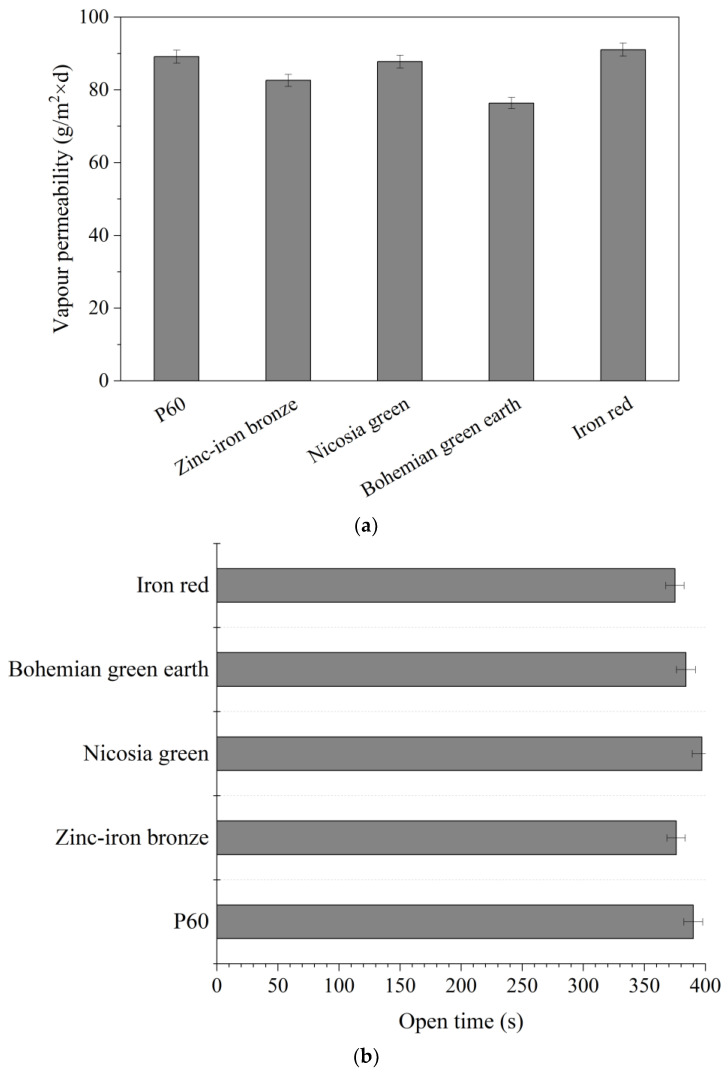
Effects of pigments on the vapor permeability (**a**) and open time (**b**) behaviour of paints.

Figure 4 presents the effects of the studied pigments on the vapor permeability and open time of the paint. As can be seen, there was a slight reduction in the vapor permeability of paint filled with Bohemian green pigment. However, the assigned class of the paint was unchanged. It should be noted that vapor permeability is often associated with lower water uptake, so improving the class for water absorption without changing the class for the vapor permeability test can be considered a success. Keeping the same level of vapor permeability means the paint is not more likely to keep water vapor inside the walls, making the walls more vulnerable to degradation. Moreover, none of the pigments had a major impact on the open time of the paint. New additives should not affect the open time of paints, which are designed to have a specific work time. Extending or reducing the open time might require changing the application instructions.

### 3.6. Contrast

Visual differences are observable by the naked eye when at least dE = 1.0. This means all of the pigments performed well in terms of mating properties. This outcome was expected as the paint contains titanium oxide white, which has an excellent mating capability. However, this result alone is not enough to ensure sufficient mating. All of the studied pigments presented satisfying mating capabilities when added to paint (Table 2). Importantly, the dry pigments performed even better than their commercial equivalents, as evidenced by lower contrast values.

### 3.7. UV Aging

**Figure 5:**
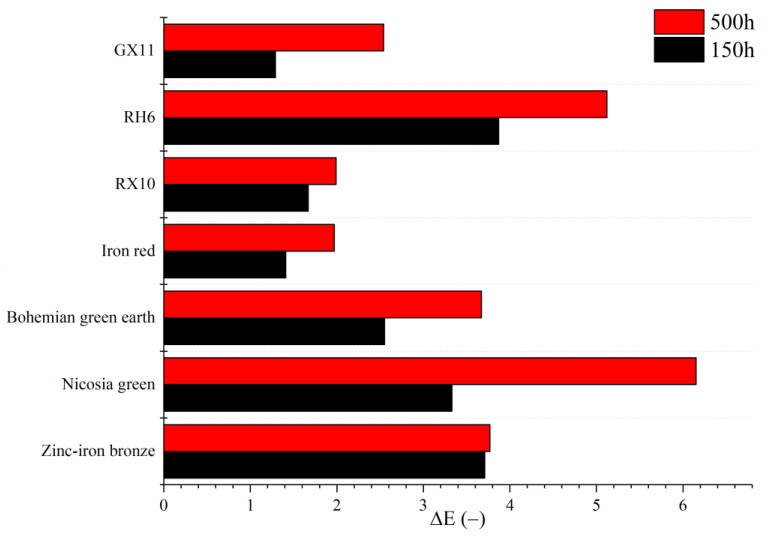
Color change (ΔE) of paint samples exposed to different durations of UV aging.

Paint samples containing the dry pigments were further exposed to long-term UV aging simulation tests. The color changes were monitored after 150 and 500 h of UV aging. The results are presented in Figure 5. Generally, the color changes (ΔE) were too slight to be noticed by the naked eye even after 500 h of testing. This indicates excellent resistance to UV light. The most satisfactory result was observed for iron-red pigment, with ΔE of less than 2 even after 500 h of aging. This result is very similar to the commercial pigment RX10, and much better than the commercial red RH6. Typically, inorganic pigments exhibit better stability against light in paints compared to their organic equivalents. However, the Bohemian green earth performed worse than its commercial counterpart, and Nicosia green showed the worst performance overall, changing color twice as much as commercial green GX11. This is unfortunate, since maintaining color is an important trait for decorative coatings, and alternatives to expensive green pigments are highly desired. The spinel–bronze pigment aged rapidly over the first 150 h. However, after reaching ΔE~4, it did not degrade much further. It is generally acceptable for pigments to lose their color properties under sunlight, although this degradation should not occur too quickly. In this case, the degradation of the samples containing dry pigments was comparable to the degradation of the samples containing commercial pigments, meaning that the dry pigment samples were acceptable in terms of UV stability.

### 3.8. New Paint Formulation (Viscosity Measurements, Storage Stability, Wet-Scrub Resistance)

Based on the results of our preliminary studies, Bohemian green earth was selected as the best performing and most promising coloring additive. The deciding factor was that it maintained the same level of viscosity as the original paint, so it did not change its application properties of the paint. Viscosity is the easiest change to observe and has the most influence on the basic properties of paint. Moreover, Bohemian green earth did not change and even improved some of the other characteristics of the paint and even improved some of them, including water absorption, making the pigment a functional additive.

After selecting the most satisfactory pigment, a new paint formulation was prepared containing 5% weight content of Bohemian green earth (see Table 3). The standard formulation was used as a reference (Table 3). Standard tests were performed on both samples. Viscosity was measured twice, immediately after sample preparation and after 24 h. Additionally, storage stability and wet-scrub resistance tests were performed. A viscosity curve was prepared using an Anton-Paar MCR102 Rheometer. 

The viscosity of the newly-formulated paint was measured according to the same method used in our preliminary studies, using a Brookfield viscometer. Measurements were taken immediately after preparation of the paint and again after 24 h to determine if any loss of viscosity had occurred. The results are shown in Table 4. As can be seen, the impact on viscosity is negligible. As our previous findings demonstrated, the pigment does not have any major effect on the basic rheological properties of the original formulation. The slight increase in viscosity over 24 h implies thixotropic behavior, which was maintained after modification.

Viscosity was measured after placing the samples in an incubator for 2 weeks at a temperature of 50 °C. The samples were observed to detect the occurrence of any sedimentation of the dispersed phase. The aim was to simulate the storage behavior of the final product. The results are presented in Table 4.

Wet-scrub resistance was tested according to the same method used in our preliminary research. The results are presented in Table 5. Samples were applied to a 300-µm substrate and left for 28 days at room temperature to dry out. The thickness of each sample was measured before and after scrubbing. Each sample was subjected to 200 cycles of scrubbing using sponges and 0.5% ABSNa solution. Scrub resistance was evaluated according to PN-EN 13300 classifications. As can be seen in Table 5, modification with Bohemian green earth did not affect the scrub resistance of the original formulation.

### 3.9. Rheological Profile of the Newly-Formulated Paint

**Figure 6:**
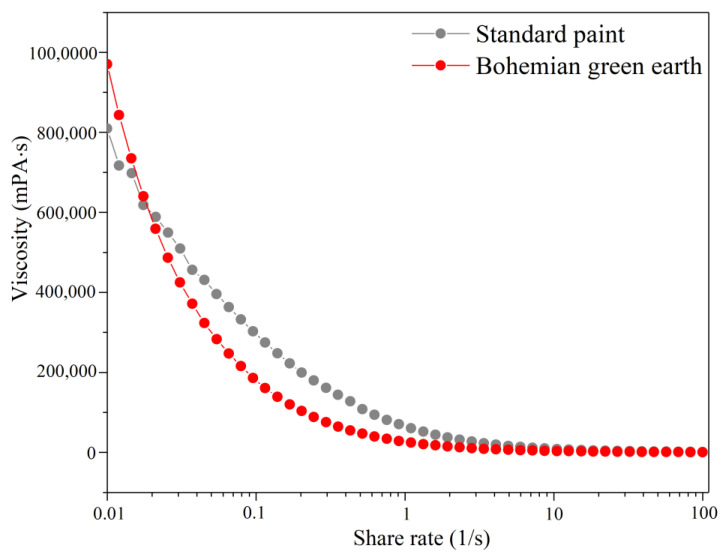
Viscosity curves for the standard paint formulation and paint with bohemian green earth.

In the last stage of the research, the rheological properties of the newly-formulated paint were evaluated using an Anton-Paar MCR102 (Graz, Austria) rotational rheometer with a plate–plate measuring system (two 25 mm diameter plates rotating in parallel with the sample placed between them). The samples were subjected to the rotational movement of the upper plate with an increasing shear rate. The rheological results were recorded on a viscosity–shear rate graph (Figure 6). The viscosity–shear rate graph can be divided into three areas. Shear rates of 0.01–1 s^−1^ correspond to storage stability. Shear rates of 1–10 s^−1^ correspond to in-can mixing and leveling. Higher shear rates correspond to brush loading and application [40].

As can be seen in Figure 6, the modified paint presents higher initial viscosity compared to the standard formulation, although it liquefies more easily at low shear-rate values (0.01–1), suggesting it might present less viscous behavior during storage. At mid-shear rates (1–10), the paints show similar viscosity, meaning they would present similar behavior while mixing in the can. However, the steeper slope in the case of the modified paint might indicate a higher tendency for sagging. Finally, in the application range (10–100) the viscosity of the samples is indistinguishable as the graphs are identical.

## 4. Conclusions

This work investigated the possible use of dry pigments as replacements for currently used colorants, even without first being liquified into pigment pastes. The best results were obtained with the addition of Bohemian green earth pigment, which had no major impact on the rheological characteristics of the original paint, meaning it can be used without being made into a pigment paste. This is a huge advantage, as green pigments tend to be the most expensive additives. Nonetheless, any change in the formulas of commercial paints, especially related to the color, brings additional costs. Each new addition to the formula should be subjected to tests to determine its impact on important properties, and its possible benefits for paint quality. 

## Figures and Tables

**Table 1:** Effects of pigments on gloss, exfoliation, and adhesion.

Pigment	Gloss at 60 [GU]	Gloss at 85 [GU]	Exfoliation [%]	Adhesion Class
Iron Red	0.6	0.5	<5	1
Bohemian green earth	1.5	0.6	<5	1
Nicosia green	1.5	0.6	0	0
Zinc–iron bronze	1.0	0.6	0	0
P60	1.4	0.5	0	0

**Table 2:** Effects of pigments on mating.

Pigment	P60	Zinc-Iron Bronze	Iron Red	Nicosia Green	Bohemian Green Earth	RX10	RH6	GX11
Contrast [dE]	0.52	0.06	0.10	0.12	0.17	0.14	0.27	0.98

**Table 3:** Reference and modified paint formula.

	Reference Paint	Modified Paint
Component	Weight Share [%]	
Water	20.0	20.0
Antifoam emulsion	0.2	0.2
Biocide	1.0	1.0
Coalescent	1.6	1.6
Titanium dioxide	3.0	3.0
**Dolomite filler**	**42.0**	**37.0**
Acrylic resin	28.0	28.0
Silicone resin	3.0	3.0
Cellulose thickener	0.5	0.5
Acrylic thickener	0.7	0.7
**Bohemian green earth**	**0.0**	**5.0**

**Table 4:** Viscosity measurements and storage stability.

	Reference Paint	Modified Paint
Initial viscosity [mPas]	15.63	15.93
Viscosity after 24 h [mPas]	16.10	16.20
Viscosity after storage [mPas]	17.03	17.13
Sedimentation	no	no

**Table 5:** Wet-scrub resistance.

	Thickness [µm]			
	Before Scrub	After Scrub	Loss	Class
Reference paint a	192.00	185.00		
Reference paint b	187.00	180.00		
Reference paint c	190.00	178.00		
Reference paint d	190.00	179.00		
Average	189.75	180.50	9.25	II
Modified paint a	197.00	190.00		
Modified paint b	205.00	189.00		
Modified paint c	190.00	184.00		
Modified paint d	191.00	190.00		
Average	195.75	188.25	7.50	II

## Appendix A

**Table A1:** Particle size distribution.

	Pigment				
Particle Radius [nm]	P60	Zinc-Iron Bronze	Iron Red	Nicosia Green	Bohemian Green Earth
Measurement 1	137.6	146.2	156.8	145.5	160.1
Measurement 2	158.0	148.7	138.6	144.0	148.9
